# Supplementary figures and images for: Nuclear receptor corepressor 1 controls regulatory T cell subset differentiation and effector function
Source: eLife. 2024 Oct 28;13:e78738. doi: 10.7554/eLife.78738 (PMC11517256; doi:10.7554/eLife.78738)

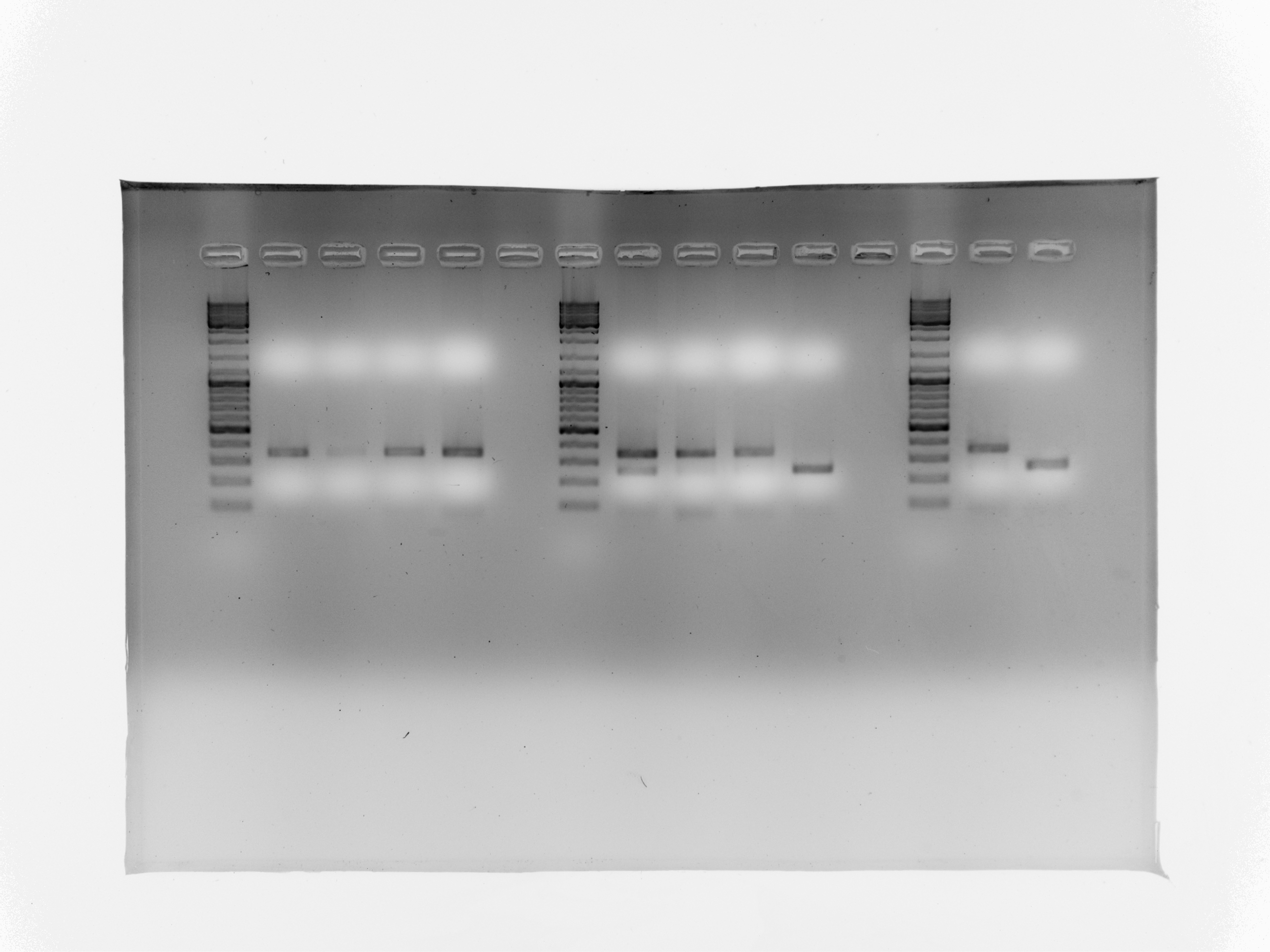

Supplement: Figure 3—figure supplement 1—source data 1. — Representative agarose gel picture showing Ncor1 deletion PCR results in WT tail samples and FACS-sorted WT CD4+ splenocytes (left panel), TCRß+, CD8+, CD4+YFP–, and CD4+YFP+ cells isolated from the spleen of NCOR1-cKOFoxp3 mice (middle panel) and CD4+YFP– and CD4+YFP+ cells isolated from the thymus (right panel) of NCOR1-cKOFoxp3 mice. Two mice were pooled for sorting. Size of floxed PCR fragment: 346bp. Size Δ fragment: 246bp. [file elife-78738-fig3-figsupp1-data1.tiff]

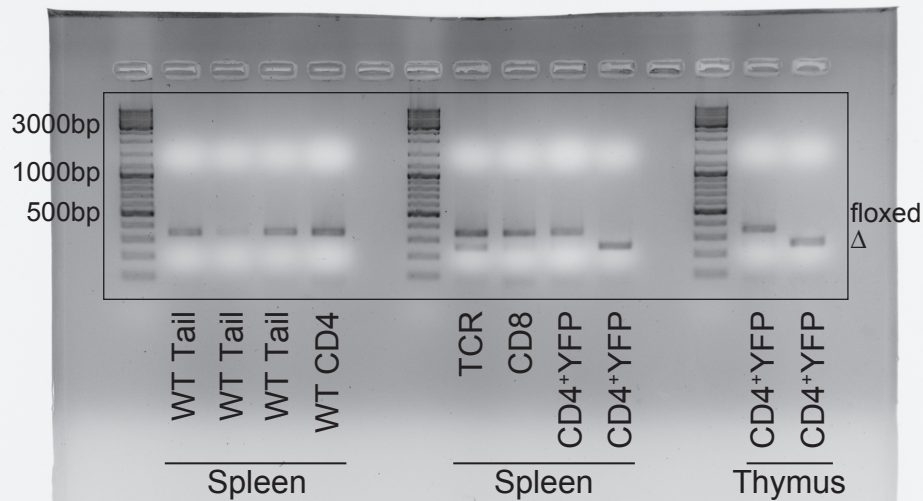

Supplement: Figure 3—figure supplement 1—source data 2. — Representative agarose gel picture showing Ncor1 deletion PCR results in wild-type (WT) tail samples and FACS-sorted WT CD4+ splenocytes (left panel), TCRß+, CD8+, CD4+YFP–, and CD4+YFP+ cells isolated from the spleen of NCOR1-cKOFoxp3 mice (middle panel) and CD4+YFP– and CD4+YFP+ cells isolated from the thymus (right panel) of NCOR1-cKOFoxp3 mice. Two mice were pooled for sorting. Size of floxed PCR fragment: 346bp. Size Δ fragment: 246bp. The black rectangle framed section in the blot shows the cropped area of the final picture in Figure 3—figure supplement 1. Band sizes are indicated on the left side of the picture. Floxed/ delta fragments are indicated on the right side of the picture. The samples are indicated in the lower part of the picture. [file elife-78738-fig3-figsupp1-data2.pdf]
